# Transient LTRE analysis reveals the demographic and trait‐mediated processes that buffer population growth

**DOI:** 10.1111/ele.13148

**Published:** 2018-09-05

**Authors:** Adriana A. Maldonado‐Chaparro, Daniel T. Blumstein, Kenneth B. Armitage, Dylan Z. Childs

**Affiliations:** ^1^ Department of Ecology and Evolutionary Biology University of California 621 Charles E. Young Drive South Los Angeles CA 90095‐1606 USA; ^2^ Department of Collective Behaviour Max Planck Institute for Ornithology Am Obstberg 1 Konstanz 78315 Germany; ^3^ Department of Biology University of Konstanz Universitätstraße 10 Konstanz 78464 Germany; ^4^ Rocky Mountain Biological Laboratory Box 519 Crested Butte CO 81224 USA; ^5^ Department of Ecology and Evolutionary Biology University of Kansas Lawrence KS 66045 USA; ^6^ Department of Animal and Plant Sciences University of Sheffield Western Bank Sheffield S10 2TN UK

**Keywords:** Environmental variation, integral projection models, life table response experiments, population dynamics, trait‐mediated effects

## Abstract

Temporal variation in environmental conditions affects population growth directly via its impact on vital rates, and indirectly through induced variation in demographic structure and phenotypic trait distributions. We currently know very little about how these processes jointly mediate population responses to their environment. To address this gap, we develop a general transient life table response experiment (LTRE) which partitions the contributions to population growth arising from variation in (1) survival and reproduction, (2) demographic structure, (3) trait values and (4) climatic drivers. We apply the LTRE to a population of yellow‐bellied marmots (*Marmota flaviventer*) to demonstrate the impact of demographic and trait‐mediated processes. Our analysis provides a new perspective on demographic buffering, which may be a more subtle phenomena than is currently assumed. The new LTRE framework presents opportunities to improve our understanding of how trait variation influences population dynamics and adaptation in stochastic environments.

## Introduction

Temporal variation in environmental conditions is a ubiquitous feature of natural systems, with potentially strong effects on vital rates such as survival and reproduction (Saether *et al*. 2000; Coulson *et al*. 2001; Mace *et al*. 2015; Koons *et al*. 2017; McDonald *et al*. 2017; Paniw *et al*. 2018). In turn, environmentally induced fluctuations in vital rates drive stochastic variation in population growth and fitness (Tuljapurkar 2010). It is well established that prevailing conditions influence population growth through their direct impact on survival and fecundity, but they also act indirectly by inducing transient changes in the population stage structure (‘demographic processes’) or ontogeny and development of cohorts (‘trait‐mediated processes’). Transient fluctuations in population structure are important when vital rates vary across different classes of individual (McDonald *et al*. 2016); for example a population may be skewed towards young, non‐reproductive individuals following a year of unusually high recruitment, leading to reduced population growth. Similar effects can also be mediated by fluctuations in the distribution of fitness‐linked traits such as body size (Benton *et al*. 2006); for example if poor environmental conditions decrease the body mass of a cohort, this may reduce their survival and reproduction in future years (van Benthem *et al*. 2017). The population level consequences of such effects are difficult to tease apart because their impacts are expressed over multiple years and may involve more than one life‐history process (Beckerman *et al*. 2003; Van de Pol *et al*. 2006; Monaghan 2008).

All else equal, variation in population growth reduces the stochastic fitness of a population (Tuljapurkar 1982), leading to selection for physiological, behavioural or life‐history strategies that minimise variation in population growth. This observation has led to the development of the demographic buffering hypothesis (Pfister 1998). In its most general form, this hypothesis predicts that the vital rates to which population growth is most sensitive will be selected to become the least variable, leading to a negative correlation between the population growth rate sensitivity and temporal variance of vital rate parameters. Despite technical challenges, ample empirical evidence has accumulated to support this general prediction (Pfister 1998; Gaillard *et al*. 2000; McDonald *et al*. 2017), though it is far from universal (Jongejans *et al*. 2010). However, correlational evidence of this kind does not consider the full array of indirect demographic and trait‐mediated processes that may mediate demographic buffering. While the impact on population growth of a particular pathway can be large, different environmental drivers and pathways may act antagonistically to buffer populations from the total effect of the environment (Tuljapurkar *et al*. 2009). Moreover, studying the total (co)variance of vital rates ignores the possibility that demographic buffering may evolve in response to particular axes of environmental variation, such as temperature or precipitation.

The interplay between fluctuating environmental conditions, transient fluctuations in age/stage structure, trait variation and realised vital rates have only recently begun to be evaluated (Dahlgren & Ehrlén 2009; Brooks *et al*. 2016). Structured population projection models – including matrix projection models (MPM; Caswell 2001) and integral projection models (IPM; Ellner *et al*. 2016) – are central to this endeavour (Dahlgren & Ehrlén 2011). Dissecting the dependence of vital rates on environmental conditions requires observations across a range of conditions to identify relevant covariates and characterise functional relationships (Frederiksen *et al*. 2008; Morris *et al*. 2008). Estimating these relationships is challenging when multiple vital rates are temporally variable or multiple environmental drivers influence the same process. These challenges are most easily addressed within an IPM framework, where information about the trait dynamics and vital rates, along with the environmental dependence of these associations, is completely described by a small set of time‐varying regression parameters (Merow *et al*. 2014; Rees *et al*. 2014; Ellner *et al*. 2016). Each parameter is associated with a specific vital rate that can affect the population in predictable ways. For example the parameters governing state‐dependent survival and fecundity directly determine population growth rate in a given year. However, they may also influence the demographic structure of the population, which will impact on population growth in future years.

With a parameterised model in hand, the impact of different sources of variation on annual population growth rate can be quantified using a life table response experiment (LTRE) analysis (Caswell 2001). The goal of a *random* LTRE is to decompose the variance in growth rate into contributions arising from the temporal (co)variances of model parameters (Brault & Caswell 1993; Caswell 2001). The standard random LTRE is based on the (linear) first order Taylor approximation:

(1) $$\text{Var}\left({\tilde{\lambda }}_{t}\right)\approx \sum_{i=1}^{n}\sum_{j=1}^{n}\text{Cov}\left(\theta_{\mathrm{it}},\theta_{\mathrm{jt}}\right)s_{i}s_{j}$$

where $\tilde{\lambda_{t}}$ is the asymptotic growth rate associated with the year‐specific projection kernel, Cov(θ_*it*_, θ_*jt*_) is the temporal covariance of θ_*it*_ and θ_*jt*_ and *s*_*i*_ is the eigenvalue sensitivity for parameter θ_*i*_ calculated in the mean kernel (Ellner *et al*. 2016). In the presence of small environmental variation, the functional dependence of $\tilde{\lambda_{t}}$ on each time‐varying parameter is approximately linear. In reality, the environmental variance is often large and demographic parameters are nonlinearly related to population growth rate. To address this challenge, Rees and Ellner (Rees & Ellner 2009) introduced a Monte Carlo approach to random LTREs that uses a statistical model to partition the variance of $\tilde{\lambda_{t}}$. However, this approach assumes that the population remains close to the stable population structure implied by each projection kernel, which means it does not account for the impacts of transient variation in trait or (st)age structure.

We show how to construct a Monte Carlo *transient* LTRE to address these restrictions. Instead of working with year‐specific asymptotic growth rates ($\tilde{\lambda_{t}}$), the LTRE partitions variance in the *realised* annual population growth rate at time *t* (λ_*t*_) into contributions from the model parameters at different time lags. This separates the contribution of direct effects that play out immediately from the indirect delayed drivers of variation that act through changes in population structure. We then show how to extend the analysis to partition the component contributions from different environmental (e.g. climatic) factors to these variance components. Capitalising on 37 years (1976–2012) of individual‐based data from a population of yellow‐bellied marmots (*Marmota flaviventer*), we parameterise an environmentally driven, stochastic Integral Projection Model with body mass‐ and stage‐dependent demographic rates. Individual vital rates and body mass in marmots are strongly influenced by environmental conditions (Van Vuren & Armitage 1991; Ozgul *et al*. 2010; Maldonado‐Chaparro *et al*. 2015), particularly by winter duration and summer rainfall (Armitage 1994, 2014). However, the drivers of variation in population growth have not been quantified, and nothing is known about the potential role of demographic buffering in this system. We use the transient LTRE to quantify the impact of these drivers and investigate the potential for demographic buffering in this population.

## Monte Carlo LTREs

### Monte Carlo random LTREs

A random LTRE assumes that the time‐varying demographic rates and trait transitions are drawn from a joint probability distribution. The goal is then to partition the variance in annual population growth rate associated with the parameters that govern these processes, *θ*_*it*_. In an IPM framework, random LTREs are most informative when constructed with respect to the ‘low level’ regression parameters that define the vital rate functions – typically the intercepts of generalised linear models, such that $E\left(vital\;rate_{\mathrm{it}}\right)=g^{-1}\left(\theta_{\mathrm{it}}+\cdots \right)$, where *g* ^−1^ is the nonlinear inverse link function. Monte Carlo random LTRE analysis proceeds as follows:

Step 1 Simulate a long sequence of realisations of the time‐varying parameters θ_*it*_, for *t *=* *1, …, *t*_*max*_) from their joint distribution.
Step 2 Construct the year‐specific IPM kernel from the sampled parameters and compute the asymptotic population growth rate implied by that kernel, $\tilde{\lambda_{t}}$.
Step 3 Fit a predictive statistical model to $\tilde{\lambda_{t}}$ using the θ_*it*_ as predictors, and use term‐wise predictions from the fitted model to partition the variance explained by θ_*it*_.

Any predictive modelling framework could be used in the last step (Ellner *et al*. 2016). Using a linear model (LM), the predictive model is ${\tilde{\lambda }}_{t}=\beta_{0}+\sum_{i=1}^{n}\beta_{i}\theta_{\mathrm{it}}+\varepsilon_{t}$, where the β_*i*_ are regression slopes associated that capture the estimated (partial) effect of parameter *i* on $\tilde{\lambda_{t}}$. The corresponding variance decomposition is then:

(2) $$\text{Var}\left({\tilde{\lambda }}_{t}\right)\approx \sum_{i=1}^{n}\sum_{j=1}^{n}\text{Cov}\left(\theta_{\mathrm{it}},\theta_{\mathrm{jt}}\right)\beta_{i}\beta_{j}+\text{Var}\left(\varepsilon_{t}\right),$$

where $\tilde{\lambda_{t}}$ is the asymptotic growth rate associated with the year‐specific projection kernel, Cov(θ_*it*_, θ_*jt*_) is the temporal covariance of θ_*it*_ and θ_*jt*_ and Var(ε_*t*_) is the unexplained variance. One advantage of using the Monte Carlo random approach is that the linear model effectively averages parameter sensitivities over their range of variation (Rees & Ellner 2009). The difference between the estimated β_*i*_ and the sensitivities in eqn (1) arises from nonlinearities in the relationship between the asymptotic growth rate and model parameters. Nonlinearities can be accommodated by adopting a more flexible model such as a generalised additive model (GAM). The model is then ${\tilde{\lambda }}_{t}=\beta_{0}+\sum_{i=1}^{n}\eta_{i}\left(\theta_{\mathrm{it}}\right)+\varepsilon_{t}$, where η_*i*_() is a nonlinear smooth function estimated with the GAM. The corresponding variance decomposition becomes:

(3) $$\text{Var}\left({\tilde{\lambda }}_{t}\right)\approx \sum_{i=1}^{n}\sum_{j=1}^{n}\text{Cov}\left(\eta_{i}\left(\theta_{\mathrm{it}}\right),\eta_{j}\left(\theta_{\mathrm{jt}}\right)\right)+\text{Var}\left(\varepsilon_{t}\right),$$

where Cov(η_*i*_(θ_*it*_), *η*_*j*_(θ_*jt*_)) is now the contribution induced through the nonlinear impact of the parameters on $\tilde{\lambda_{t}}$, and Var(ε_*t*_) is the unexplained variance.

### Monte Carlo transient LTREs

The Monte Carlo random LTRE introduced by Rees and Ellner (Rees & Ellner 2009) effectively assumes that the population is always close to the stable structure implied by each kernel – the $\tilde{\lambda_{t}}$ are the leading eigenvalues of each kernel. Two simple modifications make it possible to partition contributions arising from transient fluctuations in the stage structure and trait distributions. First, we replace the eigenvalues with the realised values of λ_*t*_ from a full simulation of the population dynamics. These are a function of the current kernel and the current state distribution at each iteration. In a stationary environment, this distribution is a result of the prior sequence of time‐varying parameters, up to some maximum lag. The second modification is to include lagged parameters in the predictive model for the realised λ_*t*_ to explain the variance induced by delayed effects. For example the GAM model underpinning the Monte Carlo LTRE becomes $\lambda_{t}=\beta_{0}+\sum_{k=0}^{l}\sum_{i=1}^{n}\eta_{\mathrm{ik}}\left(\theta_{i(t-k)}\right)+\varepsilon_{t}$, where θ_*i*(*t*−*k*)_ refers to time‐varying parameter *i* at lag *k*, and $\eta_{\mathrm{ik}}\left(\theta_{i(t-k)}\right)$ is the corresponding estimated smooth function. The variance decomposition is then:

(4) $$\text{Var}\left(\lambda_{t}\right)\approx \sum_{k=0}^{l}\sum_{i=1}^{n}\sum_{j=1}^{n}\text{Cov}\left(\eta_{\mathrm{ik}}\left(\theta_{i(t-k)}\right),\eta_{\mathrm{jk}}\left(\theta_{j(t-k)}\right)\right)+\text{Var}\left(\varepsilon_{t}\right).$$

This decomposition assumes temporal autocorrelation in the environmental process is negligible (i.e. the θ_*it*_ are *iid*), because it only considers covariances across parameters at shared lags. Autocorrelation could be accommodated by including covariances across parameters at different lags, resulting in a modified version of eqn (4) with four summations across parameters and lags.

### Decomposing environmental drivers of variation

An environmentally driven IPM can be constructed by including environmental covariates in one or more of the underpinning regression models. For example a varying intercept (generalised) linear model takes the form $E\left(vital\;rate_{\mathrm{it}}\right)=g^{-1}\left(\theta_{\mathrm{it}}+\cdots \right)$, where θ_*it*_ is now defined as $\theta_{\mathrm{it}}=\phi_{\mathrm{it}}+\sum_{p=1}^{m}\alpha_{\mathrm{pi}}e_{\mathrm{pt}}$; the *e*_*pt*_ are the values of the time‐varying environmental covariates, α_*pi*_ are coefficients that capture the effect of these covariates on the vital rate and ϕ_*it*_ are time‐varying intercepts that capture additional variation not explained by the covariates. Under this model for environmental effects, the linear approximation for the variance of $\tilde{\lambda_{t}}$ expands to:

(5) $$\text{Var}\left({\tilde{\lambda }}_{t}\right)\approx \sum_{i=1}^{n}\sum_{j=1}^{n}\left[\text{Cov}\left(\phi_{\mathrm{it}},\phi_{\mathrm{jt}}\right)+\sum_{p=1}^{m}\sum_{q=1}^{m}\alpha_{\mathrm{pi}}\alpha_{\mathrm{qj}}\text{Cov}\left(e_{\mathrm{pt}},e_{\mathrm{qt}}\right)\right]s_{i}s_{j},$$

where *s*_*i*_ is again the eigenvalue sensitivity for parameter θ_*i*_ calculated from the mean kernel, and $\text{Cov}\left(e_{\mathrm{pt}},e_{\mathrm{qt}}\right)$ and $\text{Cov}\left(\phi_{\mathrm{it}},\phi_{\mathrm{it}}\right)$ are the temporal covariances between the environmental covariates and the unexplained variance terms respectively. Three factors determine the net contribution of environmental covariates to $\left(\lambda_{t}\right)$: the magnitude of their (co)variances, $\text{Cov}\left(e_{\mathrm{pt}},e_{\mathrm{qt}}\right)$, the strength of their effects on the vital rate, α_*pi*_, and the sensitivity of population growth rate to θ_*it*_, given by *s*_*i*_.

Constructing the corresponding transient Monte Carlo LTRE will be challenging if the dependences of λ_*t*_ on θ_*it*_ is nonlinear because this induces a hierarchy of effects that cannot be additively partitioned. In principle, it is possible to construct such a decomposition using ideas from nonlinear path analysis (Scheiner *et al*. 2000), though this will make the implementation and interpretation of the decomposition significantly more complicated. However, it is straightforward to construct a transient Monte Carlo LTRE when the dependences of λ_*t*_ on θ_*it*_ is approximately linear. If the environmental effects are also linear the variance decomposition is directly analogous to eqn (6):

(6) $$\text{Var}\left(\lambda_{t}\right)\approx \sum_{k=1}^{l}\sum_{i=1}^{n}\sum_{j=1}^{n}\left[\text{Cov}\left(\phi_{i\left(t-k\right)},\phi_{j\left(t-k\right)}\right)+\sum_{p=1}^{m}\sum_{q=1}^{m}\alpha_{\mathrm{pi}}\alpha_{\mathrm{qj}}\text{Cov}\left(e_{p(t-k)},e_{q(t-k)}\right)\right]\beta_{i}\beta_{j}+\text{Var}\left(\varepsilon_{t}\right),$$

where the β_*i*_ coefficients are estimated from a transient Monte Carlo LTRE with respect to only θ_*it*_, i.e. the total effect of the unexplained variation (ϕ_*it*_) and the environment (*e*_*pt*_). If the environmental effects act nonlinearly, the corresponding covariance term will instead include the transformed terms, similar to eqn (4).

### Validating and interpreting the LTRE

An advantage a model‐based LTRE is that regression diagnostics can be used to investigate model adequacy (Rees & Ellner 2009). We advocate a pragmatic approach, whereby the performance of possible candidate models is evaluated in terms of *R* ^2^, regression diagnostics and interpretability. For example we assumed that the individual terms act additively and defined our transient LTRE in terms of $\text{Var}\left(\lambda_{t}\right)$, in order to remain consistent with the usual linear approximation. However, it may usually be more appropriate to decompose the variance of log(λ_*t*_), because the kernel is a nonlinear function of the low‐level time‐varying parameters and the state‐trait distribution at time *t* depends non‐additively on the prior sequence of environments (Fox & Gurevitch 2000). We also only included univariate terms, which assume that interactive effects between parameters are negligible. However, higher order terms can easily be incorporated and evaluated in terms of the additional variance explained.

Once a suitable model has been identified, graphical summaries of the η functions (or β slopes) and the distributions of θ_*it*_ provide a simple way to understand how each term contributes to population growth. The former represent sensitivity surfaces that account for the immediate (*k* = 1) or delayed (*k* > 1) impact on log(λ_*t*_), conditional on the other terms in the model and averaged over the full range of variation in population structure induced by the stochastic environment. Finally, the contributions from each term in the (co)variance term in the LTRE can be rescaled by the total variance of λ_*t*_ to summarise their relative contributions. For example using the GAM version of the LTRE in eqn (4), the scaled contributions are:

(7) $$\frac{\text{Cov}(\eta_{\mathrm{ik}}\left(\theta_{i(t-k)}\right),\eta_{\mathrm{jk}}\left(\theta_{j(t-k)}\right))}{\text{Var}\left(\lambda_{t}\right)}$$

## Application to Yellow‐Bellied Marmots

### Modelling

Our population of yellow‐bellied marmots at the Rocky Mountain Biological Laboratory (RMBL; Colorado, USA; 38°57′ N, 106°59′ W), has been studied since 1962 (Armitage 2014). We used data collected from all females with known age, trapped between 1976 and 2012, because detailed local weather data were available for this period. For every female individual in the study we estimated its body mass in two census points of the growing season: 1 June and 31 August as described in Maldonado‐Chaparro *et al*. (2017). We used a set of three climatic variables obtained from the RMBL weather station (106°59.588′ N, 38°773′ W at 2900 m). Winter mean temperature and spring mean temperature correspond to the average daily mean temperature in °C calculated from 1 November of the previous year to 31 March of the current year, and from 1 April to 31 May of the current year respectively; snow‐free date (SF) represents the day of the year when no snow remained on the ground at the weather station.

We describe the marmot life cycle using a post‐reproductive census (Fig. S1), from the end of the active season (31 August) prior to the main mortality period (hibernation). To describe the mass‐ and stage‐dependent demography we fitted (generalised) linear mixed effect models ((G)LMM; Appendix S1) to describe the dependence of mortality and reproductive probability on body mass, the mass‐dependent growth dynamic changes across development due to ontogeny and to phenotypic plasticity and the body mass probability distribution of juveniles weaned. The regression models describing the mass‐ and stage‐dependent demography showed a positive relationship between: (1) the probability of survival, the probability of reproduction and weaned litter size with body mass; (2) offspring body mass and mother's body mass in August; (3) growth and body mass in August the previous year and body mass in June and (4) body mass in June and body mass in August (Fig. S2; parameter values and fitted functions in Table 1).

**Table 1:** Average parameter estimates describing the association between 31 August mass (*z*) (cube root transformed) and demographic and trait transition rates

Function	Model	Fitted GLM
Survival	logit(s)	−2.229 + 0.163*z* − 0.068 *T*_*winter*_ * *+ 0.0002*T*_*spring*_ − 0.0001*SF*
Reproduction	logit(p_b_)	−2.605 + 0.225*z *+ 0.162*T*_*winter*_ * *+ 0.033*T*_*spring*_ * *+ 0.001*SF*
Recruitment	log(b)	−0.557 + 0.096*z *+ 0.002*T*_*winter*_ − 0.004*T*_*spring*_ − 0.0005*SF*
Ontogenetic growth_w_	H_0_ H_1_	μ_0_ = 1.975 + 0.651*z *+ 0.056*T*_*winter*_ **+ 0.021*T*_*spring*_ **+ 0.013*SF* μ_1_ = μ_0_ − 0.742 + 0.064*z* σ^2^ = 0.572
Ontogenetic growth_s_	H'_0_ H'_1_	μ_0_ = 10.946 + 0.360z* + 0.065*T*_*winter*_ **+ 0.024*T*_*spring*_ **+ 0.0005*SF* μ_1_ = μ_0_ − 0.612* *+ 0.041*z* σ^2^ = 0.611
Recruitment mass	C_0_	7.788* *+ 0.237*z *+ 0.002*T*_*winter*_ **+ 0.107*T*_*spring*_ − 0.003*SF* σ^2^ = 0.771

Notes

All functions included cube root body mass and the climatic variables winter temperature (*T*_*winter*_), spring temperature (*T*_*spring*_) and snow‐free date (SF) as fixed effects and year as a random effect. The functions ontogenetic growth in winter (H), ontogenetic growth in summer (H') additionally included age and the interaction between age and body mass in the fixed effects. All functions were modelled using generalised linear mixed models using the specified error structure. The coefficients presented correspond to the averaged estimates, μ_0_ corresponds to average growth of an individual of age *a* (0 or 1), and σ^2^ the variance in the ontogenetic growth and number of individuals of mass *z* recruited on year. The data fitted to the models correspond to female, yellow‐bellied marmots of all ages, from a population in and around the Rocky Mountain Biological Laboratory collected between 1976 and 2012. In the table the following conventions were used: survival (s), reproduction (p_b_), recruitment (b) and recruitment mass (C_0_).

We used an information‐theoretic model‐averaging approach (Burnham & Anderson 2002) to quantify the climate effects on each demographic and trait transition function. The climate model selection suggested differences in the effects of environmental factors on each of the vital rates (Table S1). Variation in winter temperature was associated with changes in both survival and reproductive probability, but recruitment (i.e. the number of individuals that a non‐juvenile individual weans) did not show a response to any of the environmental variables. Mass changes during the winter and summer were mainly associated with snow‐free date and winter temperature respectively. Whereas, offspring mass in August was mainly influenced by spring temperature.

On the basis of these demographic and trait transition functions, we constructed a density‐independent, stage‐structured, stochastic IPM (Rees & Ellner 2009; Rees *et al*. 2014). The model describes the temporal dynamics of the population density and the distribution of body mass (z) in juvenile (J) and non‐juvenile (A) stages. Our model assumes that: (1) the demography of individuals are determined only by their body size and stage; (2) birth and death rates in the population is density‐independent (Oli & Armitage 2003; Armitage 2014); (3) all individuals the population expressed the same plasticity in response to environmental change; (4) the body size growth dynamics of individuals are captured by two functions describing the growth of juveniles and non‐juveniles and (5) the probability of an individual being alive and available for capture at time *t* given the individual was alive at *t *− 1 (i.e. apparent survival) reflects the true survival probability of an individual (i.e. the probability of being alive at time *t* given the individual was alive at *t *− 1), although it underestimates the true survival due to emigration (Ozgul *et al*. 2009). We used a two‐step Monte Carlo resampling approach (Metcalf *et al*. 2015) to simulate the stochastic population dynamics while preserving the between‐year correlations in vital rates. The structure of the IPM and the Monte Carlo resampling approach is described in Appendix S2.

### Results: Transient LTRE

We decomposed the variance in annual population growth using both linear (LM‐) and generalised additive (GAM‐) transient LTREs. Residual analysis indicated that decompositions of λ_*t*_ exhibited a positive mean‐variance relationship so we chose to decompose the variance of log(λ_*t*_). We investigated the maximum lag required by comparing the *R* ^2^ of LM‐LTREs and GAM‐LTREs with different maximum lags. In both cases increasing this beyond 1 year improved the variance explained by < 1%. We adopted these ‘lag 1’ models in all further analyses. The LM‐LTRE and GAM‐LTRE explained 97.5 and 99.5% of variance of log(λ_*t*_) respectively. The LM‐LTRE analysis indicated that the variance of log(λ_*t*_) was mostly explained by the direct effect of fluctuations in survival and reproduction, with contributions of ~ 59 and ~ 37% respectively. The delayed reproduction term explained a further ~ 7% of this variance. The only covariance term with a contribution > |5%| was between survival and reproduction (−7%). The GAM‐LTRE produced qualitatively similar results: variation in survival, reproduction probability, delayed reproduction probability and the survival‐reproduction covariance represented 65, 37, 9 and 8% of the variance of log(λ_*t*_) respectively. We also fitted all possible pairwise smooths to the GAM‐LTRE to evaluate whether interactions between the vital rate parameters were important. We found that each of these terms represented ≪ 1% of the variance of log(λ_*t*_), meaning these could be excluded.

**Figure 1:**
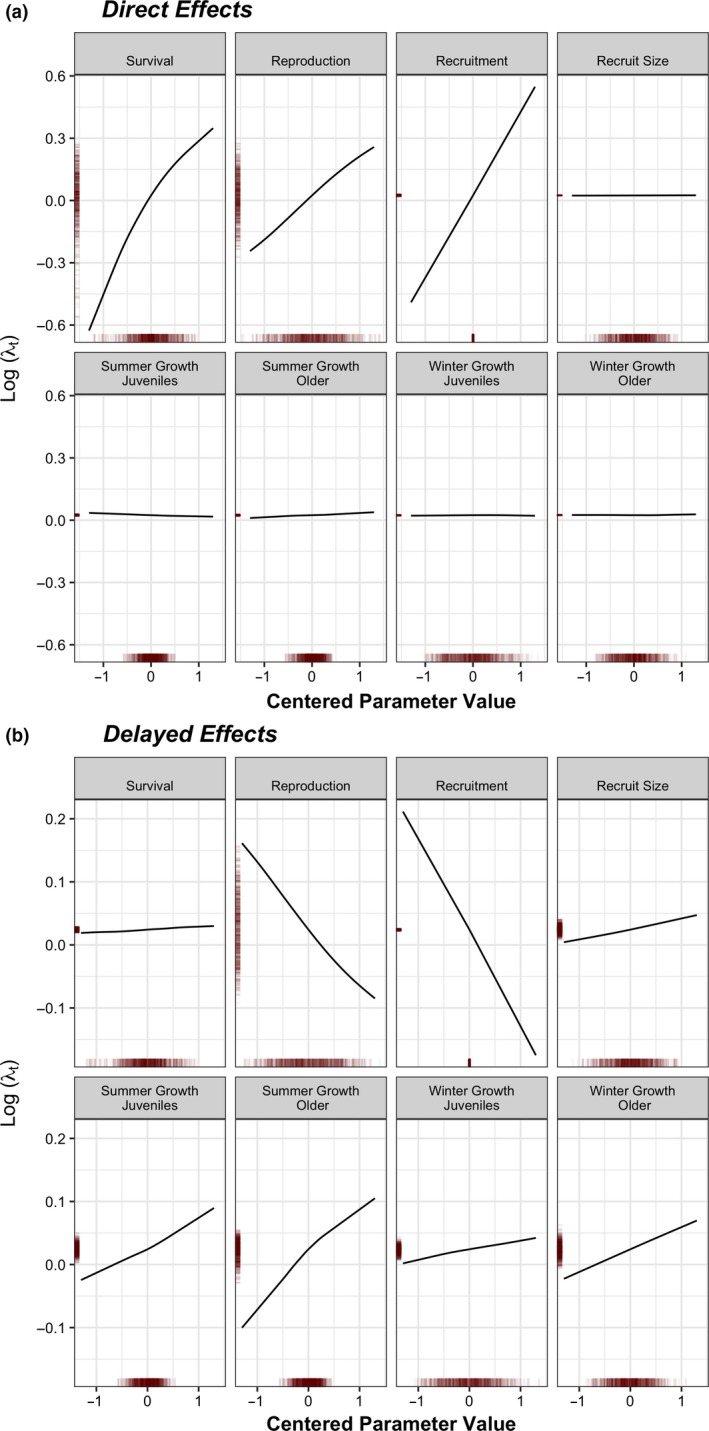
Sensitivity surface illustrating the contribution to the population growth rate, log(λ_*t*_). (a) Illustrates the direct (*k *=* *0) contributions from each of the vital rate parameters, θ_*it*_; and (b) Illustrates the delayed (*k *=* *1) contributions from each of the delayed (‘lag 1’) vital rate parameters θ_*i*(*t−*1)_. Vital rate parameters (*x*‐axis) were mean‐centred to facilitate comparisons. Rugs on the *x*‐axis and *y*‐axis illustrate the distribution of the θ_*it*_ and the distribution of the log(λ_*t*_) contribution respectively.

Figure 1 shows the sensitivity surfaces for log(λ_*t*_) estimated from the GAM‐LTRE (i.e. the (η_*ik*_)) and a summary of the parameter variability to understand the direct (*k *=* *0)) and delayed (*k *=* *1) contributions to log(λ_*t*_) from each θ_*i*(*t−k*)_. The direct effect of survival variation is larger than reproduction primarily because its sensitivity function is steeper (the parameters have similar variance), whereas the direct effect of recruitment is negligible, despite the steep sensitivity function, because this parameter is almost time‐invariant. The direct contributions from the growth parameters are absent because a shift in body mass does not alter log(λ_*t*_) in the year it occurs. The sensitivity surfaces for the delayed effects of reproduction and recruitment show how changes in age structure influence log(λ_*t*_) (Fig. 1b). These terms have steep negative sensitivity functions because a recruitment pulse increases the proportion of non‐reproductive individuals in the future. Trait‐mediated effects occur via changes in the body mass distribution. The delayed effects attributed to the growth parameters are all positive because an increase in body mass in the current environment does not affect the population dynamics until future years. The differences between winter‐summer and juvenile‐adult growth contributions are summarised in Appendix S3.

**Figure 2:**
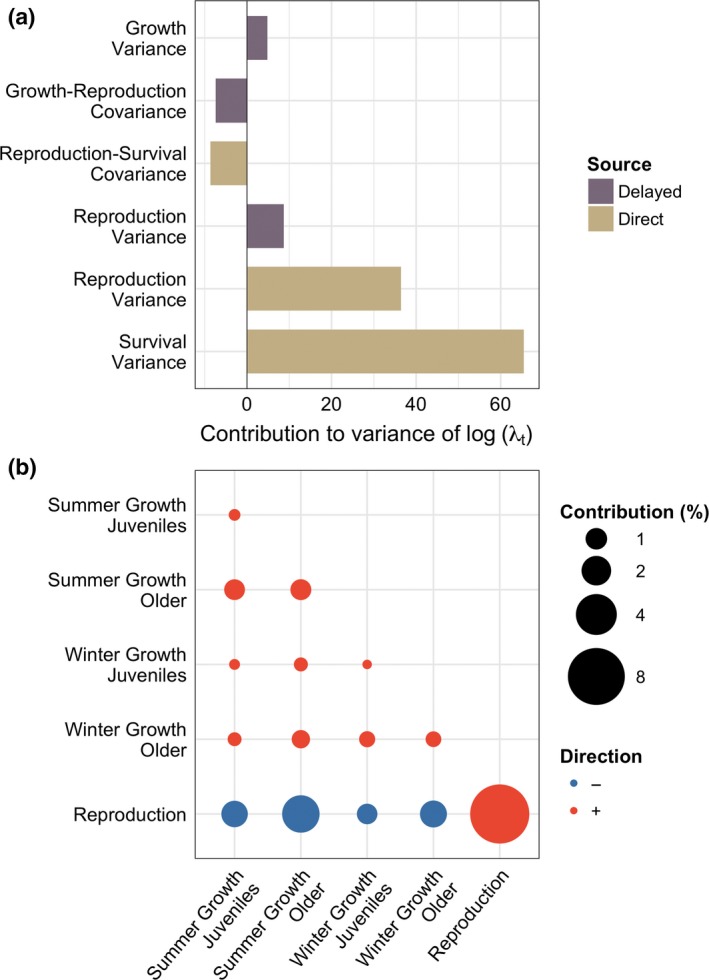
The contributions of the variance and (co)variances of the vital rates to the variance of the population growth rate, log(λ_*t*_). (a) The contribution is partitioned according to the direct (vital rate parameters, θ_*it*_), and the delayed effects (‘lag 1’ vital rate parameters, θ_*i*(*t−*1)_). Each bar indicates the scaled contribution (percentage of total variance of log(λ_*t*_)) from each parameter on the predicted value of λ_*t*_. (b) Covariation between vital rate parameters and its contribution to the predicted value of λ_*t*_. The colour of the dots illustrates the directionality of the covariation.

The individual covariance contributions were generally small. Survival and reproduction are negatively correlated (Pearson's correlation coefficient, ρ* *= −0.11), resulting in a moderate contribution of −8% to the variation in population growth (Fig. 2a). Together, the growth effects explained 6% of the variance in log(λ_*t*_) (Fig. 2a). The growth parameters all positively covary (minimum ρ* *= 0.52; Fig. 2b), and since the associated sensitivity surfaces all have positive slope, this results in a positive contribution of growth fluctuations to the variance of log(λ_*t*_). The growth and reproduction parameters positively covary (minimum ρ* *= 0.39), but the direction of the sensitivity slopes of these parameters have different signs, resulting in a negative contribution (total effect = −7%). These antagonistic effects among the growth‐reproduction (co)variances result in a very small net contribution of growth fluctuations to variation in population growth.

### Environmental drivers

**Figure 3:**
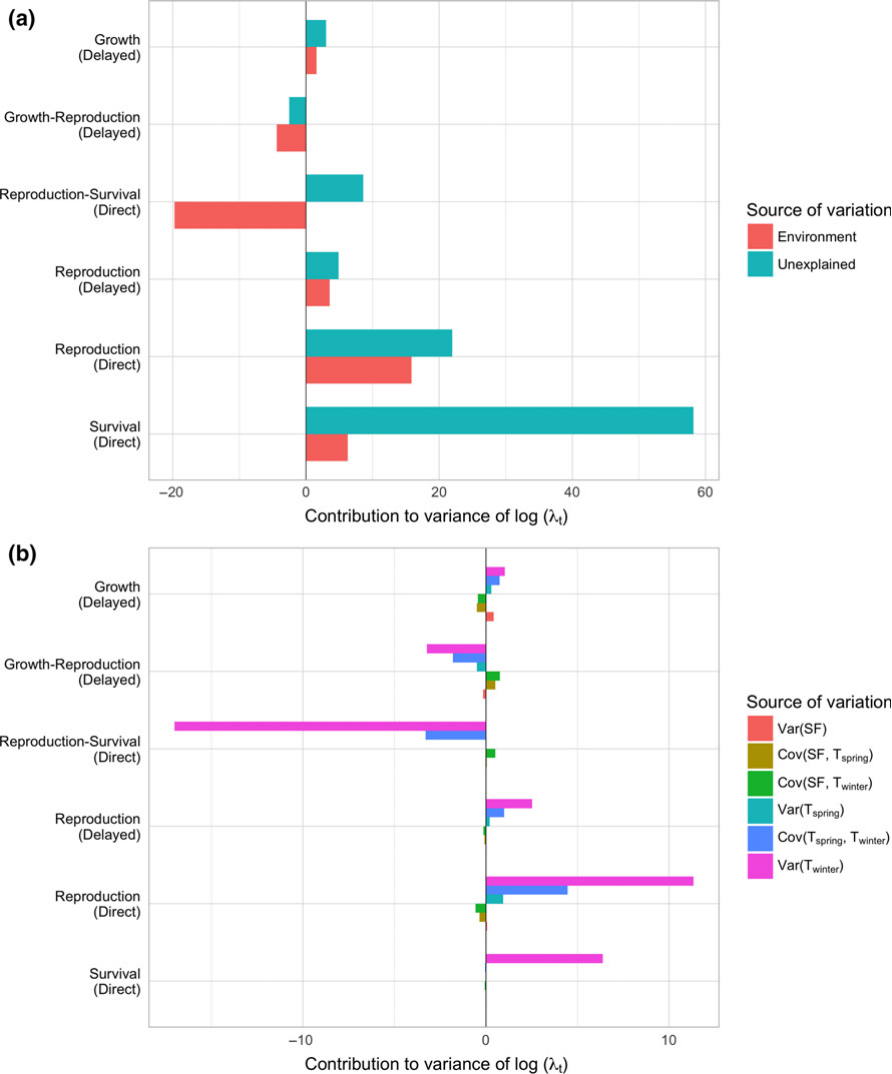
The contributions from the environmental and stochastic (unexplained) variation to the variance of the population growth rate, λ_*t*_. (a) Each bar indicates the contribution (percentage) from the six largest demographic contributors to the variance of log(λ_*t*_) decomposed into explained (environmental) and unexplained (stochastic) sources; and (b) Each bar indicates the contribution from each parameter on the predicted variance of log(λ_*t*_) decomposed into the contributions each of the environmental factors modelled.

We then used the linear LTRE to further decompose the variance of log(λ_*t*_) into sources attributed to the modelled environmental factors and the remaining unexplained stochastic variation. The effects operating through the six largest terms are summarised in Fig. 3a. The environmental effects explained relatively little variance of log(λ_*t*_) driven by the direct effect of survival fluctuations, but were similar in magnitude to the unexplained sources of variation for the remaining demographic drivers. All but one of the contributions of the environmental effects had the same direction as those due to unexplained sources. The environmental effects influencing the reproduction‐survival covariance made a large negative contribution to variance of log(λ_*t*_) (~ 20%), whereas the unexplained sources of variation increased the explained variation by 10%. The strongest contributions arising from the environmental factors (> 5% of the variance of log(λ_*t*_)) were exclusively driven by the variation in winter temperature, with a few smaller effects (1–5%) arising from the covariance between winter and spring temperature (Fig. 3b). The total absolute contribution of these effects was 59%, yet when we account for their direction the net effect is only 2%, indicating that population growth is effectively buffered from effects of temperature variation. The remaining environmental (co)variances made a negligible contribution to the variance of log(λ_*t*_).

## Discussion

Population biologists have long sought to understand how demographic processes drive temporal variation in population growth (Gaillard *et al*. 2000; Morris *et al*. 2011; Boggs & Inouye 2012), and how the environment impacts on these processes (e.g. climate; Dalgleish *et al*. 2011; Jenouvrier 2013). More recently, the role of indirect and delayed drivers of vital rate variation has begun to receive attention (e.g. Thompson & Ollason 2001). The Monte Carlo transient LTRE we adapted, serves both aims, by partitioning population growth rate variation into: (1) components due to the direct effects of environmental variation, and (2) delayed effects arising from transient fluctuations in age, stage or trait structure. Applying our model to data from a long‐term study on hibernating rodent, we quantified the effect of previously identified environmental drivers and trait‐mediated effects. This flexible approach also allowed us to accommodate nonlinear relationships between direct and indirect effects and population dynamics.

We used the Monte Carlo transient LTRE methodology to quantify sources of population growth rate variation in a wild population of marmots, a hibernating social rodent. This analysis showed that the largest components are due to direct effects of survival and reproduction, but significant delayed demographic effects are also associated with reproduction. Delayed, trait‐mediated effects of seasonal mass change made only a modest contribution to the variance of population growth rate. We also quantified the contributions arising from different environmental drivers acting through these pathways. Among the evaluated climatic drivers, variation in winter temperature was the most important factor, both through its direct influence on reproduction and survival, and through its delayed effects on reproduction and growth. Though some of the component effects of temperature were substantial in magnitude, these operate antagonistically, such that their net impact is negligible, indicating that the population is effectively buffered against temperature fluctuations during hibernation, the most important period of mortality in this population.

Populations frequently exhibit delayed demographic and life‐history responses to environmental fluctuations (Beckerman *et al*. 2002; Jenouvrier *et al*. 2005). In the marmot population, the delayed effects of reproduction (a demographic effect) and ontogenetic growth (a trait‐mediated effect) both influence population growth rate. Delayed reproductive effects arise when a pulse in reproduction skews the population to younger age classes, lowering the mean per capita reproduction the following year. This is likely to be a very general mechanism, as has been shown in Soay sheep (*Ovis aries* L., Coulson *et al*. 2001) and northern fulmar (*Fulmarus glacialis* L., Thompson & Ollason 2001), and may occur whenever vital rates differ between stage/age classes and the state distribution fluctuates over time. Delayed growth effects will occur when variation in body mass impacts demographic rates in future years. Despite the evident influence of the environment on body mass dynamics in marmots (Ozgul *et al*. 2010; Maldonado‐Chaparro *et al*. 2015), we found that interannual variation in body mass explains relatively little variation in population growth. Over‐winter changes in body mass are considerably more variable than summer mass gains. However, the former is associated with a low population growth rate sensitivity, because winter growth changes are compensated over the summer growth period. Such compensation may be common in species that have evolved to accumulate reserves over a limited window of time, such as hibernating rodents (Nicieza & Metcalfe 1997; Morgan & Metcalfe 2001).

Interannual fluctuations in climatic conditions drives significant (co)variation in demographic rates, with associated impacts on population growth (Dalgleish *et al*. 2011; Tafani *et al*. 2013; Abadi *et al*. 2017). Demographic buffering mechanisms evolve to increase stochastic fitness in the face of these fluctuations by reducing variation in population growth rate (Pfister 1998; Morris *et al*. 2008; Jongejans *et al*. 2010). Correlational evidence for demographic buffering via direct demographic effects has been accumulated from wild populations. Our study is the first to consider the role of indirect demographic and trait‐mediated pathways operating in response to specific axes of climatic variation. Because marmots are hibernating rodents, winter conditions strongly influence their energy expenditure during hibernation (Armitage *et al*. 2003). We found that although winter (and spring) temperatures impact population growth via direct and indirect effects on reproduction and survival, with an additional small contribution from arising from trait‐mediated effects, the net effect on variance in population growth rate is negligible. This indicates that the population has evolved a life‐history strategy that buffers it from this important component of environmental variation. Demographic buffering is achieved through a suite of pathways that include both direct and delayed, demographic and trait‐mediated effects, though the latter contributions are small. Few studies have found support for demographic buffering in wild populations (Morris & Doak 2004; Rotella *et al*. 2012), in part because it is methodologically challenging to (Jäkäläniemi *et al*. 2012; Bjorkvoll *et al*. 2016), a limitation our LTRE methodology overcomes.

Temporal covariation between demographic rates can be an important secondary driver of variation in population growth (Coulson *et al*. 2005; Ezard *et al*. 2006; Jongejans *et al*. 2010; Morris *et al*. 2011). A negative covariance between vital rates is expected to reduce variability because the direct sensitivities of population growth rate to vital rates such as survival, growth and reproduction are positive (Brault & Caswell 1993; Doak *et al*. 2005). In our system, the most important covariance contributions arise from the direct effects of survival and reproduction, and the delayed effects of reproduction and growth, both of which reduce the variance of population growth. The direct survival‐reproduction contribution is driven by a negative covariance between these rates. The delayed reproduction‐growth contribution represents a novel pathway, whereby *positive* covariance contributes to a *reduction* in population growth variation, because the sensitivity of population growth to the delayed (i.e. lagged) reproduction term is negative. This represents a general mechanism which can only be identified once delayed effects of reproduction have been quantified.

The marmot population model led to a simple transient LTRE in which only a single lag was required to capture the impact of the past environments. This is a consequence of the marmot's fast, compensatory growth and their short juvenile period, which lead to fast‐decaying trait‐mediated and demographic effects. We predict that in populations where the pace of growth is slow and the juvenile period is extended, these effects will decay more slowly and longer parameter lags will need to be considered. In addition, we found that a purely additive model was able to explain > 99% of the variance in population growth (i.e. interactive effects were negligible). This is unlikely to be a general result. For example when the slope of the trait‐dependent survival function varies over time, there will be fluctuations in size‐selection, and the realised state distribution will depend on the prior sequence of both growth and survival parameters. Such effects can be captured by including interaction terms that incorporate lagged values of these two classes of parameters.

The transient LTRE analysis we developed in this study complements recent efforts to understand how unstable population structure influences population growth (Koons *et al*. 2016, 2017). The strength of our approach is that it provides mechanistic insights into the joint impact of demographic and trait‐mediated processes on population growth, though it assumes a stationary environment and requires development of an appropriate regression model to capture the relevant effects. We have shown, that although body mass is an important fitness‐linked trait in the marmot system (Ozgul *et al*. 2010), its role in driving population fluctuations is relatively small, and that the population is buffered against impacts of temperature fluctuations. Importantly, our analysis demonstrates that demographic buffering may be a more complex phenomenon than is currently assumed. Due to the growing body of evidence for climatic impacts on species traits (Gardner *et al*. 2011; Sheridan & Bickford 2011), there is a need to understand the role played by trait‐mediated and demographic responses in other systems to evaluate their wider impact on population processes. Future work should identify the conditions under which we expect to observe substantial demographic and trait‐mediated effects, and determine the extent to which buffering of population growth against the effects climatic drivers is common in nature – such information will provide valuable insights for predicting population responses to future environmental change.

## Authorship

AM‐C, DTB and DZC conceptualised the study, AM‐C, DTB and KBA collected the data, DZC designed the analytical methods, AM‐C and DZC analysed the data, all authors discussed results, AM‐C wrote the first draft of the manuscript and all authors contributed to the final manuscript.

## Data Accessibility Statement

Data available from https://doi.org/10.5281/zenodo.1401488
