# Supplementary material for: Transient LTRE analysis reveals the demographic and trait‐mediated processes that buffer population growth
Source: Ecol Lett. 2018 Sep 5;21(11):1693–703. doi: 10.1111/ele.13148 (PMC6849557; doi:10.1111/ele.13148)
Supplement: Supplementary file 1 [file ELE-21-1693-s001.docx]

**Supporting Information**

**Appendix S1: *Demographic data and model fitting***

In our population, individuals are live-trapped each year, individually identified by a numbered ear tag the first time they are captured, and body mass is measured multiple times during the summer. Female reproductive success was calculated based on whether they were observed to wean a litter. The number of weaned offspring was estimated as the number female juveniles that emerged from the natal burrow (offspring were assigned to a female either observationally (1976 to 2000—Armitage 2014) or using a molecular genealogy (2001-2012—Blumstein *et al.* 2009)). These data were used to fit generalized linear mixed effects models (GLMM; described below) to describe: 1) annual probability of survival, $s^{\left( t \right)}(z)$, that describes the probability of an individual of mass *z* survives to the next census; 2) annual probability of reproduction, $p_{b}^{\left( t \right)}(z)$, which describes the probability a non-juvenile individual of mass *z* reproduces the following year given they survive, and 3) recruitment, $b^{\left( t \right)}(z)$, which describes the number of individuals that a non-juvenile individual of mass *z* weans in time *t* + 1 (i.e., weaned litter size).

We then fitted linear mixed effects models (LMM; described below) to describe the mass-dependent growth dynamic changes across development due to ontogeny and to phenotypic plasticity. We divided growth into two steps and fitted separate functions to describe the two key periods of body mass change in the marmot life cycle. These define a pair of growth kernels, that together define the probability density function of body masses mass *z′* in August in year *t* + 1 of an individual of stage class *S* and mass *z*. The winter growth kernel, $H_{S}^{\left( t \right)}\left( z^{*},z \right)$, describes the body mass loss from August (*z*) in year *t* to June ($z^{*}$) in year *t* + 1. The summer growth kernel, $H_{S}^{*\left( t \right)}\left( z^{'},z^{*} \right)$, describes the body mass gain from June ($z^{*}$) to August ($z'$) in the year *t* + 1. The first function describes body mass loss due to hibernation and late spring activity. The second describes the summer active season body mass gain following emergence from hibernation. Finally, we parameterized an offspring body mass kernel, $C_{0}^{\left( t \right)}\left( z^{'},z \right)$, to describe the body mass probability distribution of juveniles weaned observed at time *t* + 1, conditional on the mother’s August body mass in the previous year, *t*.

We used an information-theoretic model-averaging approach (Burnham & Anderson 2002) to quantify the climate effects on each demographic rate and trait transition function. We fitted a set of (G)LMMs, for each of the demographic and transition rates as a function of body mass and climatic variables (i.e., spring temperature, winter temperature, and bare ground date) as fixed effects, and year as a random effect to account for additional temporal environmental variation. We excluded observations from non-reproductive individuals (< 2 years old) from the reproduction models. The two growth functions included the interaction between stage and body mass. We started with a full model that contained all independent variables and the corresponding random effects model (i.e., the global model), and then created a set of candidate models including nested combinations of the terms contained in the global model. All model combinations in the full set of candidate models contained a fixed effect of body mass and a random effect of and year. We assumed a binomial error distribution (logit link function) for the probability of survival function and reproduction functions, a Poisson error distribution (log link function) for the recruitment function, and a Gaussian error distribution (identity link) for the remaining functions. We ranked all the models in the full set of candidate models based on their Δ_i_ values (i.e., the difference between the model’s AIC value and the minimum AIC value in the full set) and calculated the Akaike weight (*w*_i_) for each model. We computed model-averaged parameters based on the full set of candidate models. The final averaged models were used to parameterize the IPM (summarized in Table S1). All models were fitted using the (g)lmer function from the lme4 package in R ver. 1.1-7 (Bates *et al.* 2015) in the R statistical environment v. 3.1.1 (R Core Team 2016).

**Appendix S2: *Building and validating the IPM***

***Model structure***

Using the demographic and trait transition functions, we constructed a density-independent, stage-structured, stochastic IPM (Rees & Ellner 2009; Rees *et al.* 2014). The model describes the temporal dynamics of the population density and the distribution of body mass (*z*) in juvenile (*J*) and non-juvenile (*A*) stages. The distribution of masses of a given stage (*S*) in the mass range [*z, z + dz*] at time *t* is described by a density function $n_{S}(z, t)$. The structure of the IPM is:

$n_{J}\left( z^{'}, t+1 \right)= \int_{\Omega} F_{A}^{(t)}\left( z^{'},z \right)n_{A}\left( z, t \right)dz$ (1)

$n_{A}\left( z^{'}, t+1 \right)= \int_{\Omega} P_{A}^{(t)}\left( z^{'},z \right)n_{A}\left( z, t \right)dz+\int_{\Omega} P_{J}^{(t)}\left( z^{'},z \right)n_{J}\left( z, t \right)dz$ (2)

where, $F_{A}^{(t)}\left( z^{'},z \right)$is the fecundity kernel component that describes the production of mass *z’* offspring by females of mass *z*. Juveniles (J) do not reproduce. The fecundity kernel is given by:

$F_{A}(z^{'},z)= s^{(t)}(z)p_{b}^{(t)}(z){b^{(t)}(z)C}_{0}(z^{'},z)$ (3)

where, $F_{A}(z^{'},z)$, defines the number of juveniles of mass *z’* that individuals of mass *z* can contribute to the population the following summer (i.e., individuals 1 year old in the current year can contribute to the population as 2 years old, in year t + 1). Individuals must first survive, with probability$s^{(t)}(z)$, and then reproduce with a probability, $p_{b}(z)$. The number, $b(z)$, and mass, $C_{0}(z^{'},z)$, of the new recruits depends on the mass *z* of the mother in the August of the previous year. Since the model only accounts for females, $b\left( z \right)$ corresponds to the number of female offspring per litter.

The $P_{A}^{(t)}\left( z^{'},z \right)$ and $P_{J}^{(t)}\left( z^{'},z \right)$ are the survival-growth kernels of non-juvenile and juvenile stages, respectively, which describe how individuals of mass *z* at time *t*, survive and grow to reach mass *z’* at time *t* + 1. The superscript (*t*) denotes the time-varying nature of the transition kernels. The stage *S* survival-growth kernel is:

$P_{S}^{\left( t \right)}\left( z^{'},z \right)= s^{(t)}\left( z \right)G_{S}^{\left( t \right)}\left( z^{'}, z \right)$ for *S* = J or A (4)

An individual in the population must survive from one year to the next, subject to time varying and mass dependent probability, $s^{(t)}$. The second term models stage-dependent growth, where:

$G_{S}^{\left( t \right)}\left( z^{'},z \right)= \int_{\Omega} H_{S}^{*\left( t \right)}\left( {z^{'},z}^{*} \right)H_{S}^{\left( t \right)}\left( z^{*},z \right){dz}^{*}$ (5)

Winter growth, $H_{S}^{\left( t \right)}\left( z^{*},z \right){dz}^{*}$describes the conditional probability density function of mass z* in the following June given the current mass *z* of the individual in August, and summer growth, $H_{S}^{*\left( t \right)}\left( {z^{'},z}^{*} \right)$ describes the conditional probability density function of mass *z’* in August (t) given the current mass *z*,* of the individual in June.

We numerically integrated the IPM using the ‘midpoint rule’ (Easterling *et al.* 2000) with upper and lower integration limits of 7 (343 g) and 20 (8000 g), respectively. This range was sufficient to avoid unintended eviction of individuals from the model. A mesh size of 100 was used in all the simulations, which was sufficient to calculate $\lambda_{t}$ to 5 significant figures.

***Simulating the stochastic environment***

We used a two-step Monte Carlo resampling approach to simulate the stochastic population dynamics (Metcalf *et al.* 2015). We simulated the LTRE over 100,000 years. In each year of a simulation, we first sampled a set of environmental states (spring and winter temperature, and bare ground date) from among the 37 observations available in this study. These introduce variation explained by the environmental variables. We then sampled a set of year-specific intercept parameters from among the 37 sets of estimates available. This captures the variation that was not explained by the environmental variables. The transition kernels for each annual projection were constructed by combining both sets of resampled parameters to calculate the realized intercept in the corresponding year. This resampling strategy was repeated at each iteration of the model resulting in an *iid* environment. This procedure is simple to implement but preserves the among-year correlations induced by both the sources of variation.

***Model performance***

To evaluate the predictive performance of the IPM, we projected the population dynamics using the observed sequence of climate variables and random intercepts (after discarding an initial transient period of 100 years). We then calculated the corresponding body mass distribution and the annual values of λ for each year-specific model. The annual population growth rate predicted by our model closely matched those calculated from the observed data (geometric mean annual population growth rate (λ) of the population for the period from 1976-2012 was 1.017; Figure S4). The stable mass distribution predicted by the IPM model captured the bimodal distribution of body masses for juveniles and non-juvenile marmots, although the predicted values for smaller masses slightly differ from the observed pattern (Figure S5).

**Appendix S3: *Supplementary results***

We found that the delayed effects attributed to the growth parameters are all positive, because body mass increases in the current environment do not affect the population dynamics until future years via its positive effects on survival and reproduction. Variation in winter growth of both juveniles and non-juveniles makes a smaller contribution to population variation than summer growth, even though the latter exhibits less variation. This is because the slope of summer growth function is relatively flat (Fig. S3d), meaning variation in winter growth conditions are compensated for during the summer. Body mass variation of non-juvenile individuals is more important than juveniles because they represent a larger proportion of the population (~60 % of the population), and because non-juveniles exhibit higher survival and can produce new recruits.

**Figure S1.** Life cycle diagram of yellow-bellied marmots and census points. The diagram represents the total number of individuals of mass z at time *t* and at time *t* + 1. The ontogenetic growth transition was split into two parts. The first part corresponds to the ontogenetic growth from August in year *t* to June the next year *t* + 1 (i.e., winter growth), whereas the second part represents the ontogenetic growth from June to August in the year *t* + 1 (i.e., summer growth). In this case, reproduction occurs before the census period therefore, newly born individuals are censused at the time *t* before any mortality occurs but they do suffer mortality before their next census at time t+1 at age 1. Individuals must survive with a mass-dependent probability (*s(z)*) in order to be able to reproduce (*p_b_(z)* *b(z)*) and contribute with new recruits to the population (*C_0_(z’, z)*), and to growth to the next year (*G(z*, z), G(z’, z*)*).


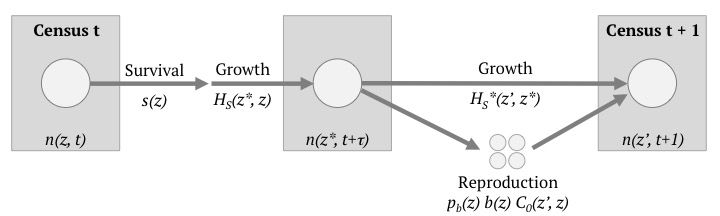


**Figure S2.** Statistical functions describing the relationship between August 31^st^ body mass (cube root) and probability of survival (a), probability of reproduction (b), and recruitment (c). Grey lines illustrate the mean response for each year in the study (from 1976 – 2012). Black continuous lines illustrate the mean population response, and rugs below the graph plot the distribution of the body mass data for non-juveniles.


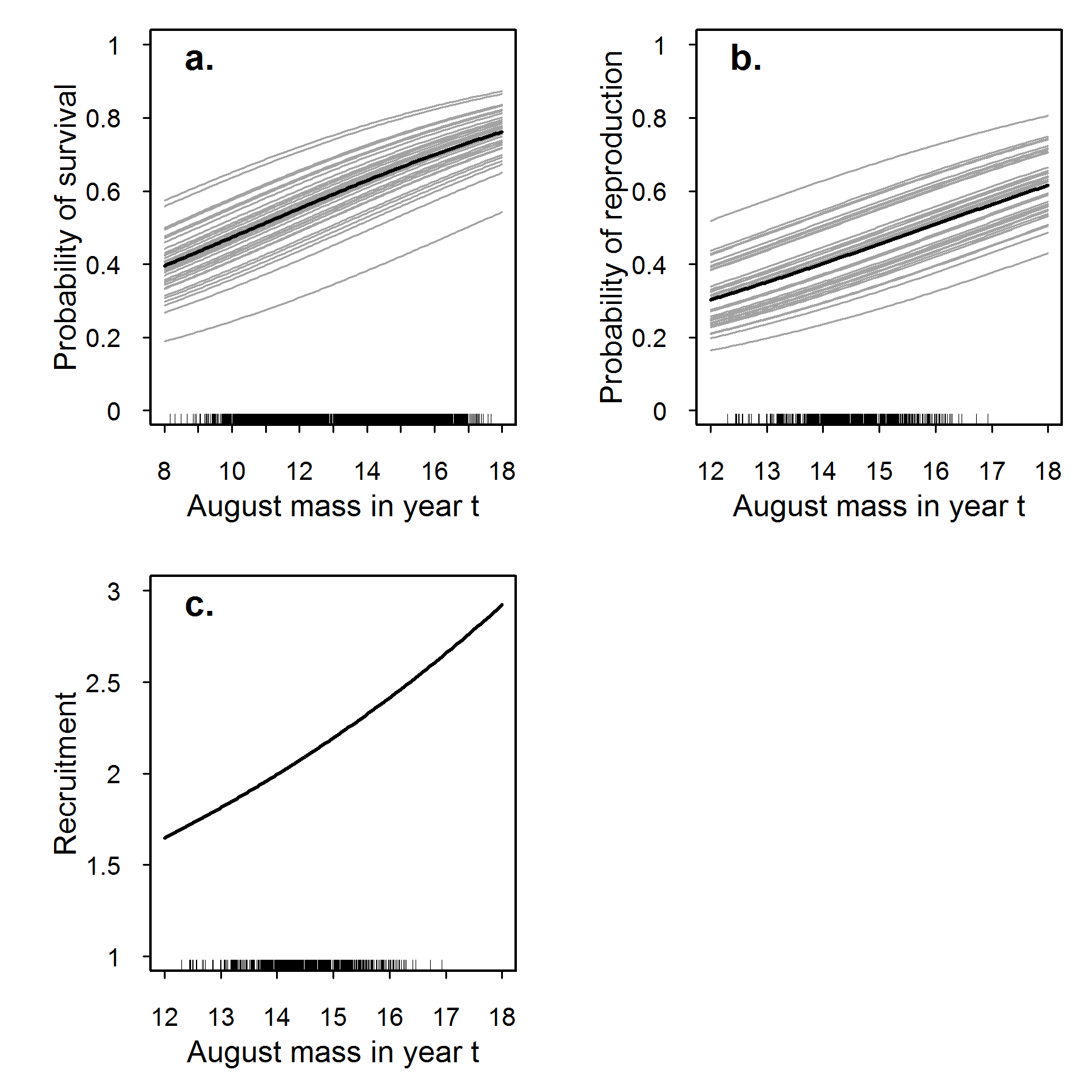


**Figure S3.** Statistical functions describing the relationship between body mass (cube root) and juvenile (a, c) and non-juveniles (b, d) individual’s growth. Upper panel illustrates winter growth, and lower panel corresponds to summer growth. Grey lines illustrate the mean response for each year in the study (from 1976 – 2012). Grey dotted lines illustrate the function y = x. Black continuous lines illustrate the mean population response, and rugs below the graph represent the distribution of the body mass data for the corresponding age class.


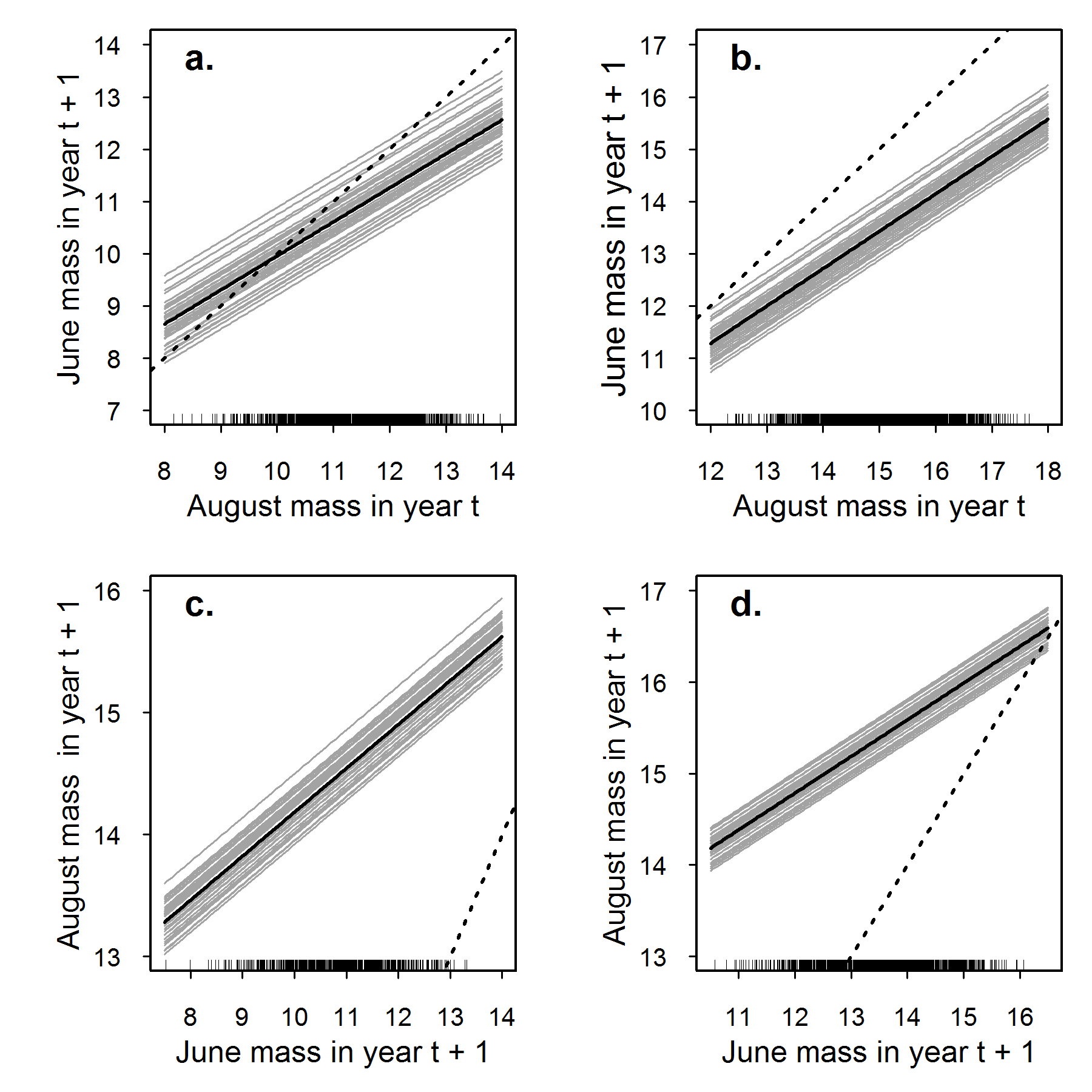


**Figure S4.** Examination of model fit – correlation between observed and simulated values of the annual change in density of yellow-bellied marmot (*Marmota flaviventer*) population over 37 years of study. a. juveniles (J), and b. non-juveniles (A). The solid line illustrates the line of best fit.


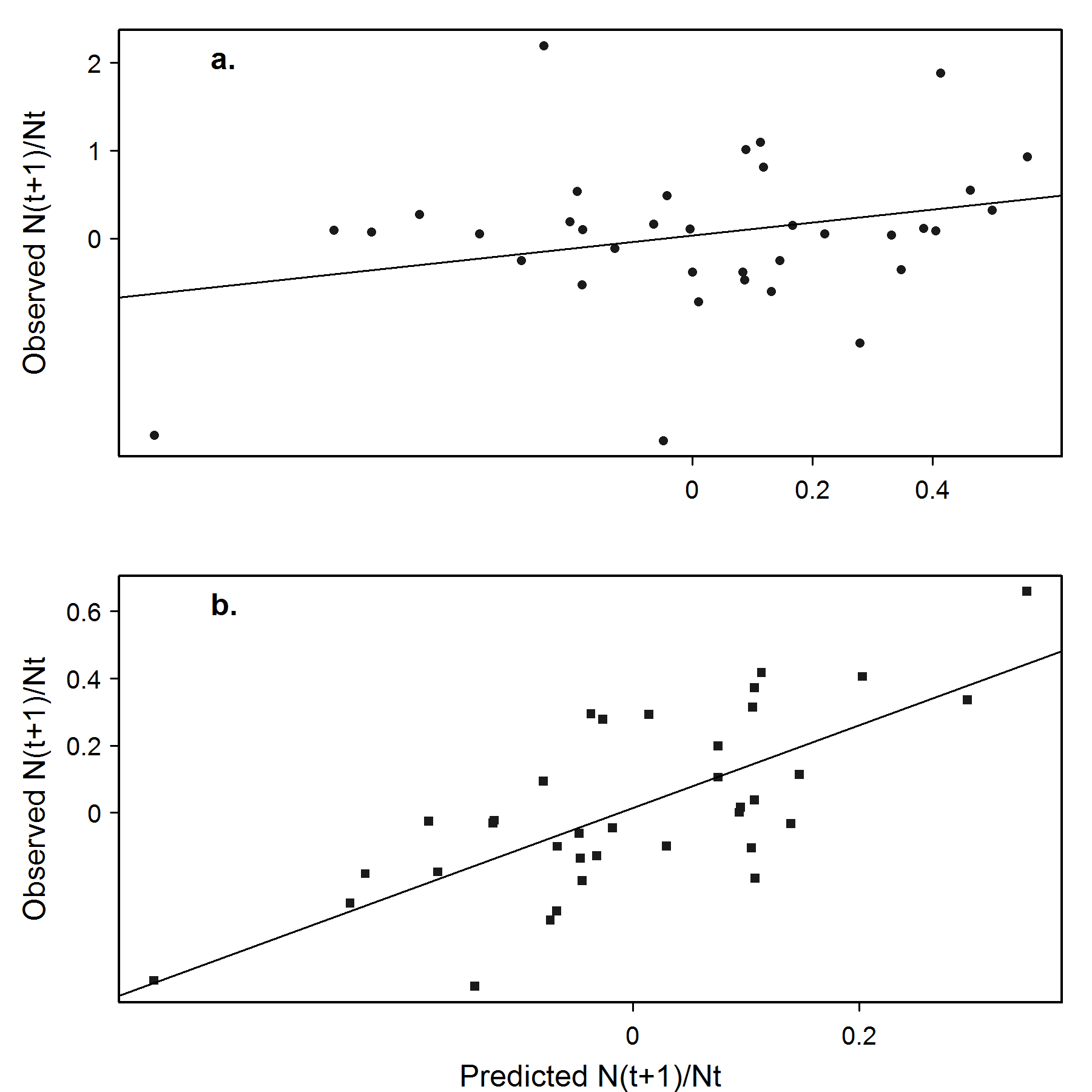


**Figure S5.** Estimated stable state distribution for body mass. Continuous line: estimated distribution of the integral projection model. Histogram: observed mass distribution.

**
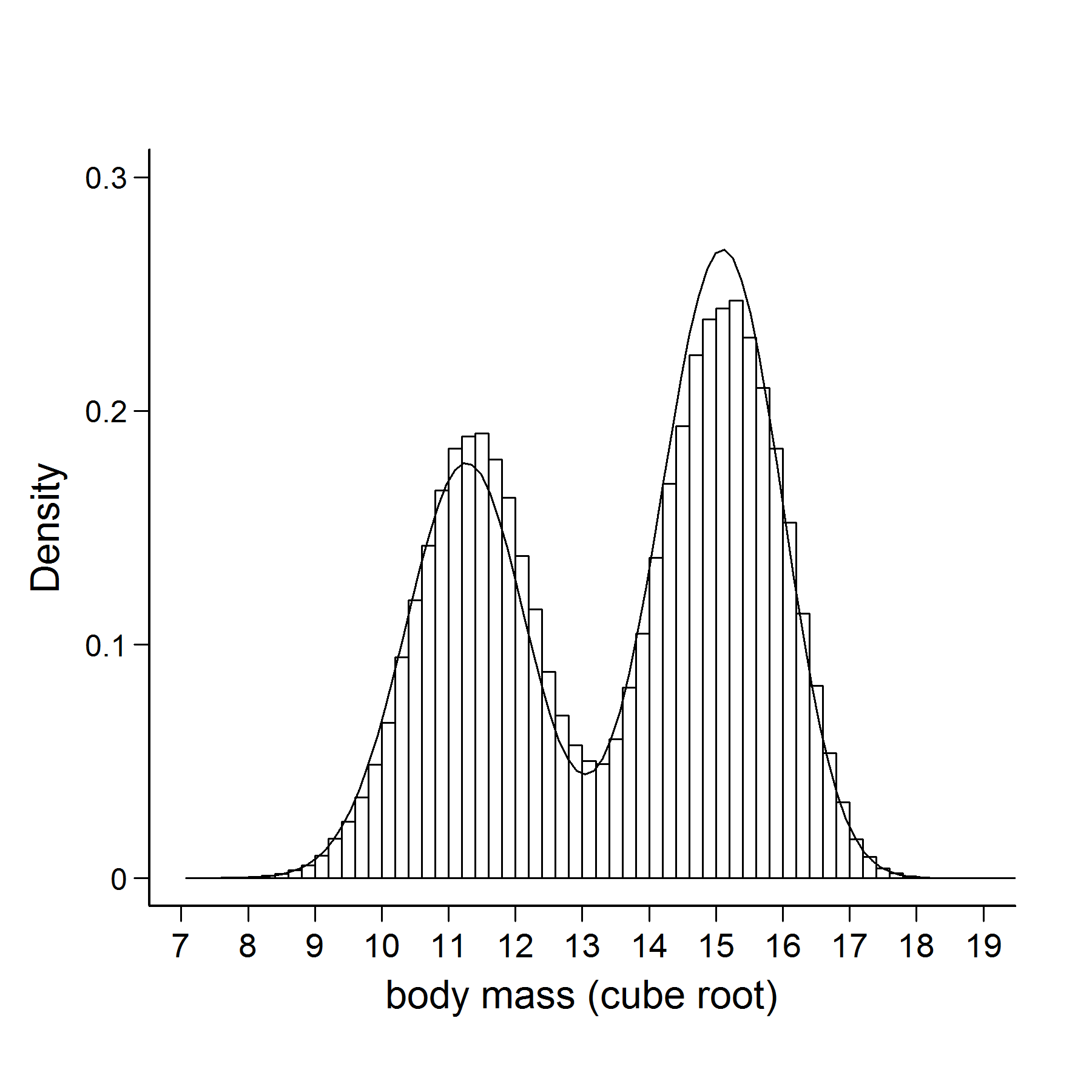
Table S1.** Summary of model selection process used to quantify the climate effects on each demographic process and trait-transitions. We show the number of models selected from the set of candidate models using Akaike information criterion (ΔAIC < 2; i.e., best fitted models). For each of the demographic process and trait-transitions we indicate the number of models in the sub-set of models that contain each environmental variable.

|  | **Number of models in the *a priori* set** | **Winter Temperature** | **Spring Temperature** | **Bare ground** |
| --- | --- | --- | --- | --- |
| **Survival (*s*(*z*))** | 4 | 3 | 1 | 1 |
| **Reproduction (*p*_b_(*z*))** | 3 | 3 | 1 | 1 |
| **Recruitment (*p*(*z*))** | 4 | 1 | 1 | 1 |
| **Winter growth (*H_S_*(*z*))** | 3 | 2 | 1 | 3 |
| **Summer growth (*H^*^_S_*(*z*))** | 3 | 3 | 2 | 1 |
| **Recruit size (*C_0_*(*z*))** | 3 | 1 | 3 | 1 |

**References:**

1.

Armitage, K.B. (2014). *Marmot biology: sociality, individual fitness, and population dynamics*. Cambridge University Press, United Kingdom.

2.

Bates, D., Machler, M., Bolker, B.M. & Walker, S.C. (2015). Fitting Linear Mixed-Effects Models Using lme4. *Journal of Statistical Software*, 67, 1-48.

3.

Blumstein, D.T., Wey, T.W. & Tang, K. (2009). A test of the social cohesion hypothesis: interactive female marmots remain at home. *Proc Biol Sci*, 276, 3007-3012.

4.

Burnham, K.P. & Anderson, D.R. (2002). *Model Selection and Multimodel Inference: A Practical Information-Theoretic Approach*. 2nd edn. Springer Science & Business Media, New York, USA.

5.

Easterling, M.R., Ellner, S.P. & Dixon, P.M. (2000). Size-specific sensitivity: applying a new structured population model. *Ecology*, 81, 694-708.

6.

Metcalf, C.J.E., Ellner, S.P., Childs, D.Z., Salguero-Gómez, R., Merow, C., McMahon, S.M. *et al.* (2015). Statistical modelling of annual variation for inference on stochastic population dynamics using Integral Projection Models. *Methods Ecol Evol*, 6, 1007-1017.

7.

R Core Team (2016). R: A language and environment for statistical computing R Foundation for Statistical Computing Vienna, Austria.

8.

Rees, M., Childs, D.Z. & Ellner, S.P. (2014). Building integral projection models: a user's guide. *J. Anim. Ecol.*, 83, 528-545.

9.

Rees, M. & Ellner, S.P. (2009). Integral projection models for populations in temporally varying environments. *Ecol. Monogr.*, 79, 575-594.
